# Linking the evolution of plant transporters to their functions

**DOI:** 10.3389/fpls.2013.00547

**Published:** 2014-01-08

**Authors:** Heven Sze, Markus Geisler, Angus S. Murphy

**Affiliations:** ^1^Cell Biology and Molecular Genetics, University of MarylandCollege Park, MD, USA; ^2^Department of Biology, Plant Biology, University of FribourgFribourg, Switzerland; ^3^Plant Science and Landscape Architecture, University of MarylandCollege Park, MD, USA

**Keywords:** phylogeny of membrane transporters, early land plants, charophyte, molecular evolution, vascular development, angiosperms, pollen, moss

In the past decade, an increasing number of plant genomes ranging from unicellular alga to trees have been completely sequenced. As the transport of water, nutrients, hormones, and metabolites in aquatic plants could differ from that of non-vascular, vascular or flowering plants, this rich resource has the potential to answer many of our most urgent questions: How did specific transporters evolve as early plants adapted to dry land? Does the evolution of transporters in monocot plants differ from that in dicots? What are the orthologs in food and energy crops of critical transporters characterized in model plants? Can we infer functions or membrane localization from phylogeny? Phylogenetic analyses of transport proteins will shed light on these questions and potentially reveal novel insights for future studies to understand the contribution of transporters in plant nutrition, stress tolerance, biomass production, as well as in signaling and development.

We invited the plant biology community to participate in this effort as ~5% of Arabidopsis thaliana (and probably other plant) genome encodes proteins with predicted transport roles. The *Arabidopsis thaliana* genome with 27 thousand protein-coding genes has over a 1000 genes classified as transporters, whereas the genomes of other flowering plants can be 2–7 times larger.

In this topic issue, several authors have examined the evolutionary history and diversification of a range of transporters and these initial results are yielding surprising insights. Some transporter families can be traced to the simplest green plants, including unicellular *Chlamydomonas reinhardtii* (Chlorophyta). Thus H^+^-coupled antiporters like NHX (Chanroj et al., 2012), CAX, MHX, CCX (Emery et al., 2012), Sultr (Takahashi et al., 2012) and primary H^+^ and Ca^2+^ pumps, such as AHA, ACA, ECA (Pedersen et al., 2012) are highly conserved in all green plants. In some cases, the gene family has diversified, for example, from 2 AHA genes in early land plant *Physcomitrella patens* to 11 genes in the flowering plant *A. thaliana*, indicating a need to regulate the proton motive force in specialized organs or cell types. In contrast, homologs of other transporter families in flowering plants were traced to a Charophyte, but not found in Chlorophyta. This history suggests that CHX co-transporters of K^+^ (Chanroj et al.), LHT amino acid transporters (Tegeder and Ward, 2012) and SUT sucrose transporter (Reinders et al., 2012) had origins in an aquatic Charophyte, and further supports Charaphytes as basal to land plants. The increase in sibling genes within each family through gene duplication and diversification, can be attributed in part to innovations in vascular tissue development (LHT in phloem) and in sexual reproduction (LHT in pollen) on land. Strikingly, CHX genes have diversified in pollen especially in dicots, suggesting roles in the delivery of sperms in pollen tubes to distant ovules. Curiously, the purine uptake permease, PUP family exists in a vascular early land plant, *Selaginella moellendorfii*, but not in a non-vascular moss (*Physcomitrella patens*) (Jelesko, 2012). It is possible that PUP genes have a role in the transport of secondary metabolites for defense and/or reproductive success in vascular plants. Early land plants, such as moss and Selaginella, possess MIP homologs similar to PIP and TIP water channels (Anderberg et al., 2012), though it is not yet clear whether aquatic Charophytes need such proteins to transport water. Chlorophytes possess MIP-like homologs though their substrate(s) may be small uncharged molecules other than water.

A striking feature is that three branches of the AtNHX family correspond to Na^+^(K^+^)/H^+^ antiporters associated with three distinct subcellular locations (plasma membrane, trans Golgi network and vacuolar membrane) in Arabidopsis (Chanroj et al., 2012). Though not all subclades of families are separated according to location, it does illustrate that finding orthologs from another species could aid in predicting function as well as membrane association (e.g., Reinders et al., 2012; Takahashi et al., 2012).

Facchinelli and Weber (2011) reviewed the evolutionary history of select metabolite transporters localized at the inner envelope of plastids. Phylogenetic studies are showing that *Arabidopsis thaliana* contributes 58% of the metabolite transporters vs. 12% from the endosymbiont cyanobacterium. The study illustrates that connecting plastid metabolism with that of the host cell is driving the evolution of metabolite transporters at the inner envelope. Authors also examine differences in phosphate transporters of photosynthetically-active plastids vs. heterotrophic plastids in non-green tissues. ATP/ADP exchangers localized to the inner envelope membrane of plastids are thought to provide ATP for reactions in heterotrophic plastids, or photosynthetic plastids during the night. However recent evidence for ATP/ADP antiporters on thylakoid membranes of Arabidopsis was surprising. Spetea et al. (2012) have traced the history of *thylakoid* ATP/ADP antiporters (TAAC) in plants and phylogenetic evidence suggests they arose once before the divergence of Chlorophyta and Streptophyta. These transporters are thought to supply ATP into the lumen for the biosynthesis and turnover of photosynthetic complexes.

Haferkamp and Schmitz-Esser (2012) re-analyzed the evolution of the Arabidopsis mitochondrial carrier family (MCF) catalyzing the transport of various substrates in mitochondria and other organelles. They found that various MCFs from Arabidopsis, yeast and human built independent clades, allowing a prediction of their biochemical function. However, some MCFs “refuse” to cluster with previously described members of subclades; their characterization presents therefore a special challenge but also a unique opportunity. Related to this work, Linka and Esser (2012) summarized the current knowledge on peroxisomal transport proteins, mainly members of the ABC transporter family known as COMATOSE (CTS) or ABCD1 shown to transport substrates for β-oxidation, and three members of the MCFs required for the import of ATP and NAD. Based on analyses of 22 fully sequenced plant genomes, evolution of these four peroxisomal transporters was an early event as they were already present in the plant genome before the cyanobacterial endosymbiont engulfment.

Carraro et al. (2012) provided a comprehensive analysis of the three major families of auxin transporters, PIN, AUX/LAX, and ABCB, from the model woody species *Populus*. They found that the array of more than 40 putative auxin transporters is reflected by a pre-existing diversity of *ABCB* genes and an expansion of *PIN* and *AUX/LAX* genes caused by genomic and segmental duplications. Interestingly, they provide further evidence that diversification of *PIN* and *AUX/LAX* genes (*Populus* owns exactly twice as many members of these families as *Arabidopsis* but a comparable number of *ABCB* genes) occurred after the origin of land plants, while the ABCB gene family diversified much earlier and long before the diversification of land plants.

Forestan et al. (2012) reanalyzed the *PIN* family in maize and confirmed that the monocot PIN family is wider and more diverged than the dicot one, though this cannot be explained simply by a gene duplication event. Interestingly, beside an increased number of Arabidopsis orthologs, maize contains three monocot-specific *PINs* (*PIN9* and *PIN10a-b*) that based on their gene expression, protein localization and auxin accumulation pattern can be partially associated with the differentiation and development of monocot-specific organs and tissues.

As more crystal structures of prokaryote and eukaryote transport proteins become available, it is now possible to examine the evolution and mechanism of plant transporters using homology modeling. Bailly et al. (2012) conducted an evolutionary analysis of ABCB substrate specificity by homology modeling of plant and mammalian ABCB1 proteins with strikingly dissimilar substrate specificities. Interestingly, they identified kingdom-specific candidate substrate binding regions within the translocation chamber formed by transmembrane domains of the ABCBs, suggesting an early evolutionary separation of plant and mammalian substrate specificity. Obviously an experimental validation of these models has highest priority. Pantoja (2012) reviewed recent mutagenesis studies to identify residues that form the ammonium transport pore and other important amino acids of AMT/MEP transporters. Zelman et al. (2012) looked at the functional domains of plant cyclic nucleotide-gated channels (CNGC) using homology modeling to get insights on the gating, the selectivity filter, and the heteromeric structure of plant CNGCs. Nussaume et al. (2011) reviewed the biological functions of plasma membrane-localized H^+^-coupled phosphate symporters of the PHT1 family, as well as their transcriptional and post-translational regulation.

Significantly, articles in this special issue demonstrate the conservation and diversification of transporters as plants evolved. The emerging patterns will allow us to predict with relative confidence the molecular and biological roles of uncharacterized genes from early land plants to energy crops, and to build a systems biology understanding of plant development, growth and survival.
